# Effect of nicotine exposure on the rate of orthodontic tooth movement: A meta-analysis based on animal studies

**DOI:** 10.1371/journal.pone.0247011

**Published:** 2021-02-17

**Authors:** Sanjay Jyothish, Athanasios E. Athanasiou, Miltiadis A. Makrygiannakis, Eleftherios G. Kaklamanos

**Affiliations:** 1 Hamdan Bin Mohammed College of Dental Medicine, Mohammed Bin Rashid University of Medicine and Health Sciences, Dubai, United Arab Emirates; 2 Department of Dentistry, European University Cyprus, Nicosia, Cyprus; 3 Department of Orthodontics, School of Dentistry, National and Kapodistrian University of Athens, Athens, Greece; University of Zurich, SWITZERLAND

## Abstract

**Background:**

Nicotine exposure has been reported to modify bone cell function and the osseous metabolism with potential effects on the rate of orthodontic tooth movement.

**Objectives:**

To systematically investigate and quantitively synthesize the most recent available evidence from animal studies regarding the effect of nicotine exposure on the rate of orthodontic tooth movement.

**Search methods:**

Unrestricted searches in 7 databases and hand searching were performed until July 2020 (PubMed, Central, Cochrane Database of Systematic Reviews, SCOPUS, Web of Science, Arab World Research Source, ProQuest Dissertations and Theses Global).

**Selection criteria:**

We searched for controlled studies on healthy animals investigating the effect of nicotine on the rate of orthodontic tooth movement.

**Data collection and analysis:**

Following study retrieval and selection, relevant data was extracted and the risk of bias was assessed using the SYRCLE’s Risk of Bias Tool. Exploratory synthesis and meta-regression were carried out using the random effects model.

**Results:**

From the initially identified records, 5 articles meeting the inclusion criteria were selected and no specific concerns regarding bias were identified. Quantitative data synthesis showed that the rate of orthodontic tooth movement in the nicotine exposed rats was higher than in the control group animals (2 weeks of force application; 0.317 mm more movement in nicotine exposed rats; 95% Confidence Interval: 0.179–0.454; p = 0.000). No effect of the concentration or the duration force application was demonstrated following exploratory meta-regression.

**Conclusion:**

Rats administered with nicotine showed accelerated rates of orthodontic tooth movement. Although, information from animal studies cannot be fully translated to human clinical scenarios, safe practice would suggest that the orthodontist should be able to identify patients exposed to nicotine and consider the possible implications for everyday clinical practice.

## Introduction

Despite recent declines, tobacco consumption globally remains considerable. According to the World Health Organization, 1.3 billion people consume tobacco in some form or the other and one quarter of the global population aged older than 15 years are current users of tobacco. Even among adolescents aged 13–15, at least 43 million use some form of tobacco [1].

Tobacco smoke has been reported to contain more than 4000 substances with important negative consequences for human health. Amongst them, nicotine is the key constituent that leads to psychological effects and long-term addiction [2]. Tobacco use is considered to be a major risk factor for pulmonary and cardiovascular diseases, over 20 different types or subtypes of cancer, glucose intolerance, dyslipidemia and many other life-threatening health conditions resulting in more than 8 million deaths every year [1–5]. Moreover, all forms of tobacco consumption and most importantly cigarette smoking have been implicated in changes to the oral mucosa leading not only to epithelial malignancy but also to periodontitis and increased tooth loss [6–8]. Even electronic nicotine delivery systems that do not contain tobacco, are considered to be harmful for the individual and unsafe for general health [1].

As different age groups can benefit from the correction of malocclusion, many individuals seeking orthodontic treatment may consume tobacco related products and be nicotine dependent. Susceptibility to smoking in orthodontic populations may be modulated by factors like family, peer influence and the social environment [9]. Furthermore, orthodontic patients may use products that contain nicotine and aim at reducing the harm from continued tobacco use [10]. Lower miniscrew success rates have been reported in smokers under orthodontic treatment [11–13]. *In vitro*, cigarette smoke has been proven to affect bond strength [14] and biofilm formation on orthodontic appliances [15].

Cigarette smoke and nicotine have been shown to affect bone cells and osseous metabolism [16–18]. Among other effects, it has been observed that nicotine upregulates the formation and differentiation of osteoclasts or osteoclast-like cells and inhibits osteogenesis, callus formation and bone mineralisation leading to decreases in bone density [16–18]. In the oral cavity, smoking has been reported to affect adversely alveolar bone height and density and is considered to be a potential risk factor for alveolar bone loss [8, 19, 20]. Kirschneck et al. [21] suggested that additional loss of periodontal bone might be expected during orthodontic treatment in smokers.

As orthodontic tooth movement involves bone resorption and formation in the alveolar processes, it may be affected by any substance implicated in the related pathways [22, 23]. Nicotine has been shown to affect bone cells and metabolism [16–18]; hence, the rate of tooth movement in orthodontic patients, who happen to be nicotine dependent, could be influenced as well. Although a previous systematic review reported qualitatively that nicotine administration accelerates the rate of orthodontic tooth movement [24], a quantitative analysis has not yet been performed.

### Objective

As research in human subjects presents significant ethical and practical limitations, the use of animal models may prove beneficial. The objective of the present meta-analysis is to systematically investigate and quantitively synthesize the most recent available evidence from animal studies regarding the effect of nicotine exposure on the rate of orthodontic tooth movement.

## Materials and methods

### Protocol development and registration

After study protocol development [25, 26] and registration (CRD42017078208), the review was carried out following relevant methodological guidelines [27–29]. As the present study was a systematic review, ethical approval was not required.

### Eligibility criteria

The Participants, Intervention, Comparison, Outcomes and Study design domains were used to formulate the eligibility criteria (S1 Table). We looked for prospective controlled studies evaluating the rate of tooth movement in animals (healthy, naïve, of any age and gender) exposed to nicotine (by any route and dosage) in comparison to non-exposed animals. All types of orthodontic mechanics were considered, and the studies had to report on the amount of tooth movement either during or after the cessation of orthodontic forces. Tooth movement could be measured with callipers or feeler gauges (directly or on casts), from histological cuts (directly on the optical microscope or from digital photos) or from various kinds of radiographs (lateral cephalometric radiographs, Cone Beam CT, micro-CT, etc.). Studies involving animals with comorbidities or dietary deficiencies, animals under medication or undergoing additional clinical interventions (i.e. tooth extraction, etc.) were excluded, as well as studies presenting qualitative assessments or not comprehensively reported quantitative assessments (i.e. without measures of central tendency and dispersion). Finally, we did not consider human, in vitro, ex-vivo or in silico studies; non-comparative studies, reviews, systematic reviews, meta-analyses and studies with less than 5 subjects per group analysed as per relevant methodological guidelines [30].

### Information sources and search strategy

Two authors (SKJ and EGK) developed detailed search strategies for each of the databases that were searched until July 2020 (MEDLINE, CENTRAL, Cochrane Systematic Reviews, Scopus, Web of Science, Arab World Research Source and ProQuest Dissertations and Theses Global database) (S2 Table). We did not impose any restrictions and we searched the reference lists of relevant studies as well.

### Study selection, data collection and data items

The same two authors assessed the retrieved records for inclusion individually and extracted from the eligible papers the following information in predetermined forms: bibliographic information, study design and eligibility; number of animals in each group and sample size calculation; age and weight of animals; nicotine administration characteristics (route, concentration), orthodontic mechanics; measurement of outcome details and reliability assessment. Numerical results were also extracted and categorized separately for each animal genus or type of mechanics used, since differences can be expected [31]. If clarifications were needed regarding the published data, or additional material was required, then attempts to contact the corresponding authors would be made. The researchers were not blinded to the identity of the authors, their institution, or the results of the research.

### Risk of bias in individual studies

Two of the authors (SKJ and MAM) assessed the risk of bias in the included studies, independently and in duplicate, using the SYRCLE’s risk of bias tool [32]. In all the above-mentioned processes disagreements were settled by discussion with AEA, but kappa statistics were not calculated [29].

### Summary measures and synthesis of results

The random effects method for meta-analysis was used to combine tooth movement summary data (Weighted Mean Difference (WMD) together with 95% Confidence Intervals (CI)), since they were expected to differ across studies due to diversity in terms of animal and intervention characteristics [33, 34]. To identify the presence and the extent of between-study heterogeneity, the overlap of 95% CI for the results of individual studies was to be inspected graphically and the I^2^ statistics were to be calculated [29]. All analyses were carried out with Comprehensive Meta-Analysis software version 3 (©2014 Biostat Inc., New Jersey, USA). Significance (a) was set at 0.05, except for 0.10 used for the heterogeneity tests [35].

### Risk of bias across studies and additional analyses

Analyses for “small-study effects” and publication bias were included in the protocol but were not performed finally due to the lack of adequate information [29]. Likewise, comparisons of the amount of movement between genera/species and studies with different orthodontic mechanics were not carried out [29]. We used meta-regression to explore whether the variation in the amount of molar mesial movement in rats was modified by the duration of tooth movement (in days) and the concentration of the administered nicotine (in mg/kg). Finally, the quality of evidence for molar mesial movement in rats after 2 weeks of force application was assessed following Guyatt et al. [36].

## Results

### Study selection

**Fig 1:**
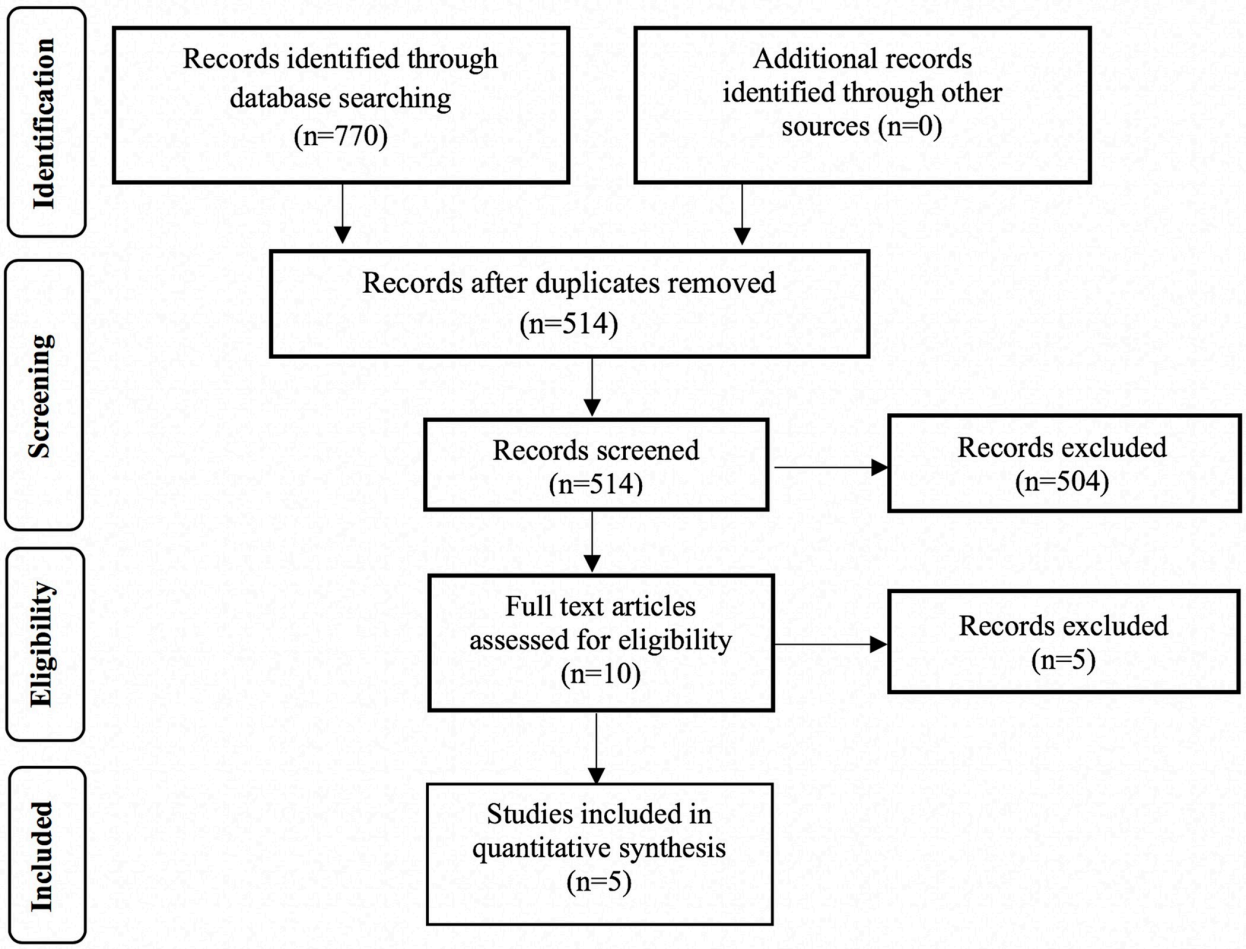
Flow of records.

The initial database search yielded 770 papers, from which 256 were excluded as duplicates and a further 504 on the basis of their title and abstract (Fig 1). Subsequently, 10 articles were reviewed in full text and five were excluded; four did not investigate the rate of tooth movement [21, 37–39] and Nagaie et al. [40] did not report comprehensively tooth movement results (the corresponding authors were contacted but no reply was received) [37–40]. Finally, 5 studies were included in the meta-analysis [41–45].

### Study characteristics

The included studies were published between 2011 and 2018 and experimented mostly on Wistar rats, except for Sodagar et al. [41] that used Sprague-Dawley and Kirschneck et al. [43] that experimented on Fischer 344 rats (Table 1). Only Kirschneck et al. [43] and Ferreira et al. [45] provided calculations on sample size; however, in the latter case the calculations were not based on the amount of tooth movement.

**Table 1:** Characteristics of the included studies.

Study	Animal characteristics	Tooth movement model | Nicotine administration	Group characteristics	Measurement methodology
**Sodagar et al.** [2011] [41]	Sprague-Dawley rats [male, adult, 250 ±20g]	NiTi CCS from R Mx I to FM [60g]	**EG**_**1**_: 8; 0.5 mg/kg; IP; 1/d	**Clinically:** interproximal feeler gauge [d 14]
		**Force application:** 14d	**EG**_**2**_: 8; 0.75 mg/kg; IP; 1/d	**Method error:** NM
		**Nicotine administration:** daily for 13d		
			**EG**_**3**_: 8; 1 mg/kg; IP; 1/d	
			**PG**: 8; 0.1 ml ns; IP; 1/d	
			**Sample size calculation:** NM	
**Bakathir et al.** [2016] [42]	Wistar rats [male, 12-week-old, 400 ±20g]	NiTi CCS from L Mx I to FM [30g]	**EG**_**1**_: 8; 0.37 mg/kg; IP; 1/d	**Clinically:** interproximal digital gauge [d 14]
		**Force application:** 14d	**EG**_**2**_: 8; 0.57 mg/kg; IP; 1/d	**Method error:** NM
		**Nicotine administration:** daily for 14d + 14d		
			**EG**_**3**_: 8; 0.93 mg/kg; IP; 1/d	
			**PG**: 8; 0.1 ml ns; IP; 1/d	
			**Sample size calculation:** NM	
**Kirschneck et al.** [2017] [43]	Fischer 344 rats [male, 6-week-old, 260 ±15g]	NiTi CCS from L Mx I to FM and SM [25g]	**EG**: 7; 1.89 mg/kg; SC; 1/d	**Radiologically:** CBCT [d 14, 28]
		**Force application:** 28d	**PG**: 7; 0.1 ml PBS; SC; 1/d	**Method error:** Yes
		**Nicotine administration:** daily for 10d + 28d*		
			**Sample size calculation:** Yes	
**Araujo et al.** [2018] [44]	Wistar rats [male, 9-week-old, 300-350g]	NiTi CCS from L Mx I to FM [25g]	**EG**: 30; 1 mg/kg; SC; 1/d ^§§^	**On dental casts:** digital caliper [d 2, 14, 28]
		**Force application:** 2, 14, 28d [10 an/group]		
			**PG**: 30; 0.1 ml ns; SC; 1/d ^§§^	**Method error:** NM
		**Nicotine administration:** 30d + 2, 14, 28d [10 an/group]		
			**Sample size calculation:** NM	
**Ferreira et al.** [2018] [45]	Wistar rats [male, 12-week-old, mean weight: 300g]	SS CCS from R Md I to FM [40g]	**EG**: 14 hemi-mandibles; 1.29 mg/kg; IN^§^	**Clinically:** digital caliper [d 7]
		**Force application:** 7d		
		**Nicotine administration:** daily for 67d + 7d [5d/week]		**Method error:** NM
			**CG**: 14 hemi-mandibles	
			**Sample size calculation:** Yes [but not for the rate of tooth movement]	

an: Animals; CCS: Closed Coil Spring; CG: Control group without sham procedure carried out; d: days; EG: Experimental group; FM: First Molar; I: Incisor; IN: Inhalation of cigarette smoke; IP: Intraperitoneally; L: Left; Md: Mandibular; Mx: Maxillary; ns: normal saline; NM: not mentioned; PBS: Phosphate Buffer Saline; PG: Placebo group with sham procedure carried out; R: Right; SC: subcutaneously; SM: Second Molars.

*Initiated 10d before force application; within the first 5d nicotine dose applied increased daily from 1/5 of the final in 1/5 increments to allow adaptation.

^§^The exposure to nicotine per day after cigarette smoke inhalation was converted to mg/kg (67, 68).

^§§^ Only the tooth movement groups, with or without nicotine administration were considered.

Nicotine in the experimental groups was being administered in concentrations ranging from 0.5 to 1.89 mg/kg; for periods of 13 to 74 days; intraperitoneally [41, 42], subcutaneously [43, 44] or by inhalation [45]. Sodagar et al. [41] and Bakathir et al. [42] investigated and reported on the effect of different nicotine concentrations. Apart from the study of Sodagar et al. [41], the other experiments included an induction period.

Orthodontic tooth movement was accomplished by attaching NiTi closed coil springs between the maxillary molars and incisors, apart from Ferreira et al. (2018) that used SS closed coil springs between the same teeth in the mandible [45]. The generated molar medializing forces ranged between 25 to 60g and the force was applied for periods between 1 and 4 weeks. Tooth movement during the application of orthodontic forces was measured clinically [41, 42, 45], on dental casts [44] or using CBCT radiographs [43]. No studies evaluating tooth movement after the cessation of the orthodontic forces were located. Apart from Kirschneck et al. [43], the remaining included studies did not report on method of error.

### Risk of bias assessment

In regard to allocation sequence generation and random housing of the animals, all studies showed an unclear risk of bias apart from Kirschneck et al. [43] that was assessed to be at low risk. Assessor blinding related risk was low for Sodagar et al. [41], Kirschneck et al. [43] and Araujo et al. [44]. Bias regarding baseline group similarity, handling of incomplete data, selective reporting and other problems was considered low. For the rest of the assessed domains the risk of bias was considered to be unclear (Table 2).

**Table 2:** Risk of bias assessment.

Signaling questions										
Study	1	2	3	4	5	6	7	8	9	10
Sodagar et al. [2011] [41]	Unclear	Low	Unclear	Unclear	Unclear	Unclear	Low	Low	Low	Low
Bakathir et al. [2016] [42]	Unclear	Low	Unclear	Unclear	Unclear	Unclear	Unclear	Low	Low	Low
Kirschneck et al. [2017] [43]	Low	Low	Unclear	Low	Unclear	Unclear	Low	Low	Low	Low
Araujo et al. [2018] [44]	Unclear	Low	Unclear	Unclear	Unclear	Unclear	Low	Low	Low	Low
Ferreira et al. [2018] [45]	Unclear	Low	Unclear	Unclear	Unclear	Unclear	Unclear	Low	Low	Low

1: Was the allocation sequence adequately generated and applied? 2: Were the groups similar at baseline or were they adjusted for confounders in the analysis? 3: Was the allocation adequately concealed? 4: Were the animals randomly house during the experiment? 5: Were the caregivers and investigators blinded to the intervention that each animal received? 6: Were animals selected at random for outcome assessment? 7: Was the outcome assessor blinded? 8: Were incomplete outcome data adequately assessed? 9: Are reports of the study free of selective outcome reporting? 10: Was the study apparently free of other problems that could result in a high risk of bias?

### Amount of tooth movement

**Fig 2:**
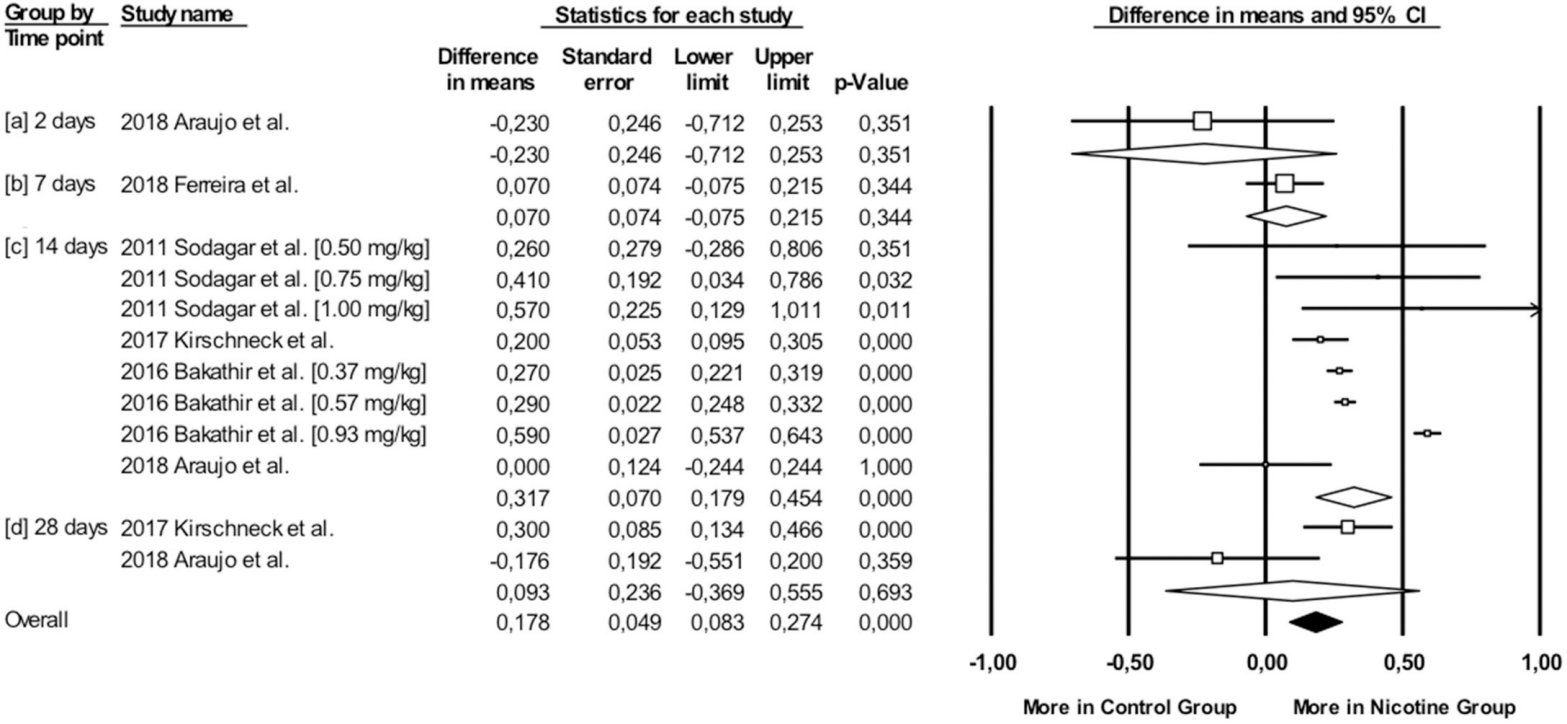
Difference in mesial molar movement between nicotine exposed or not rats, overall and at various time points.

**Fig 3:**
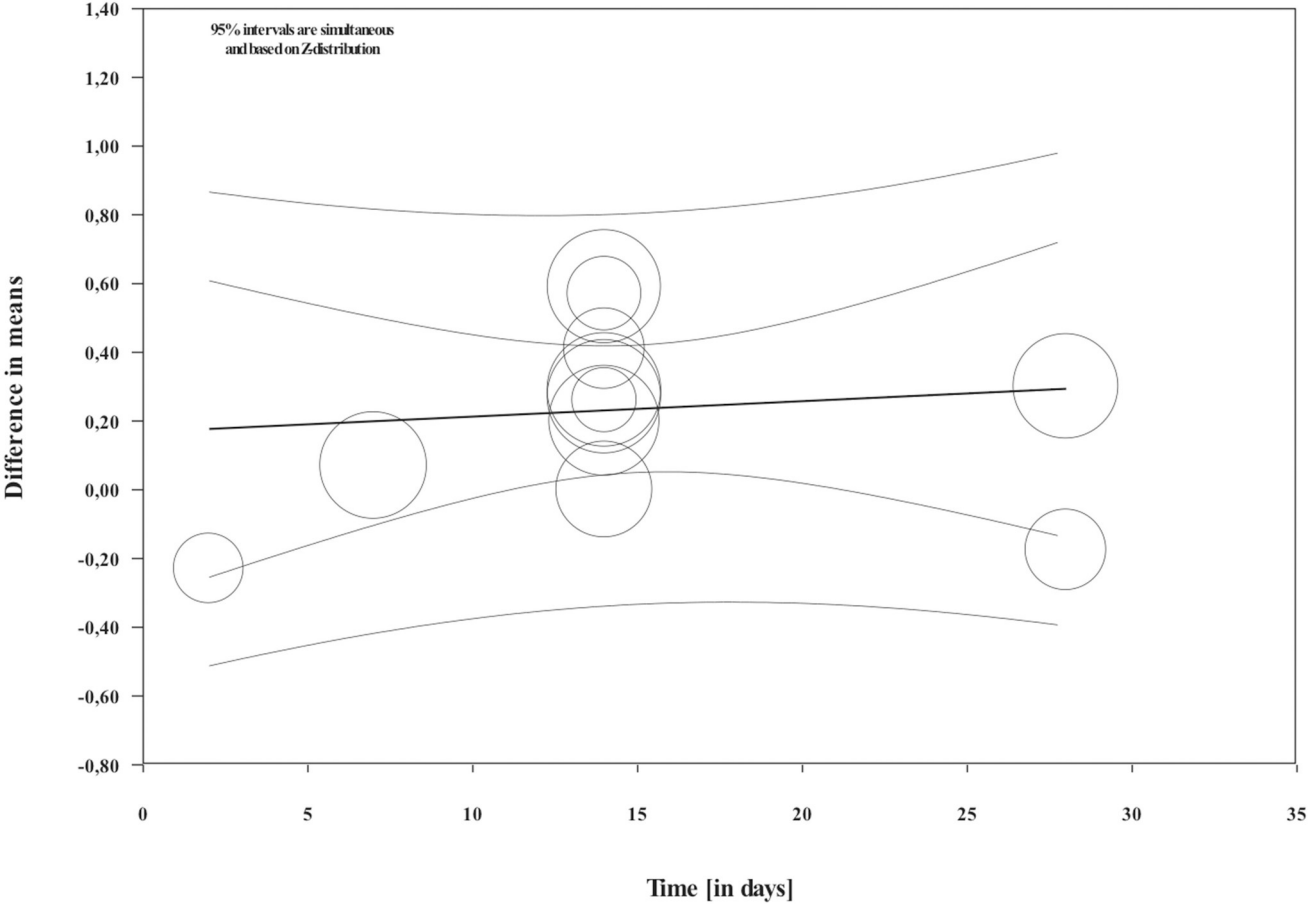
Regression scatterplot of the difference in mesial molar movement between nicotine exposed or not rats, on the predictor of the duration of force application (in days). Confidence intervals and prediction intervals are displayed (inner and outer lines around the regression line).

**Fig 4:**
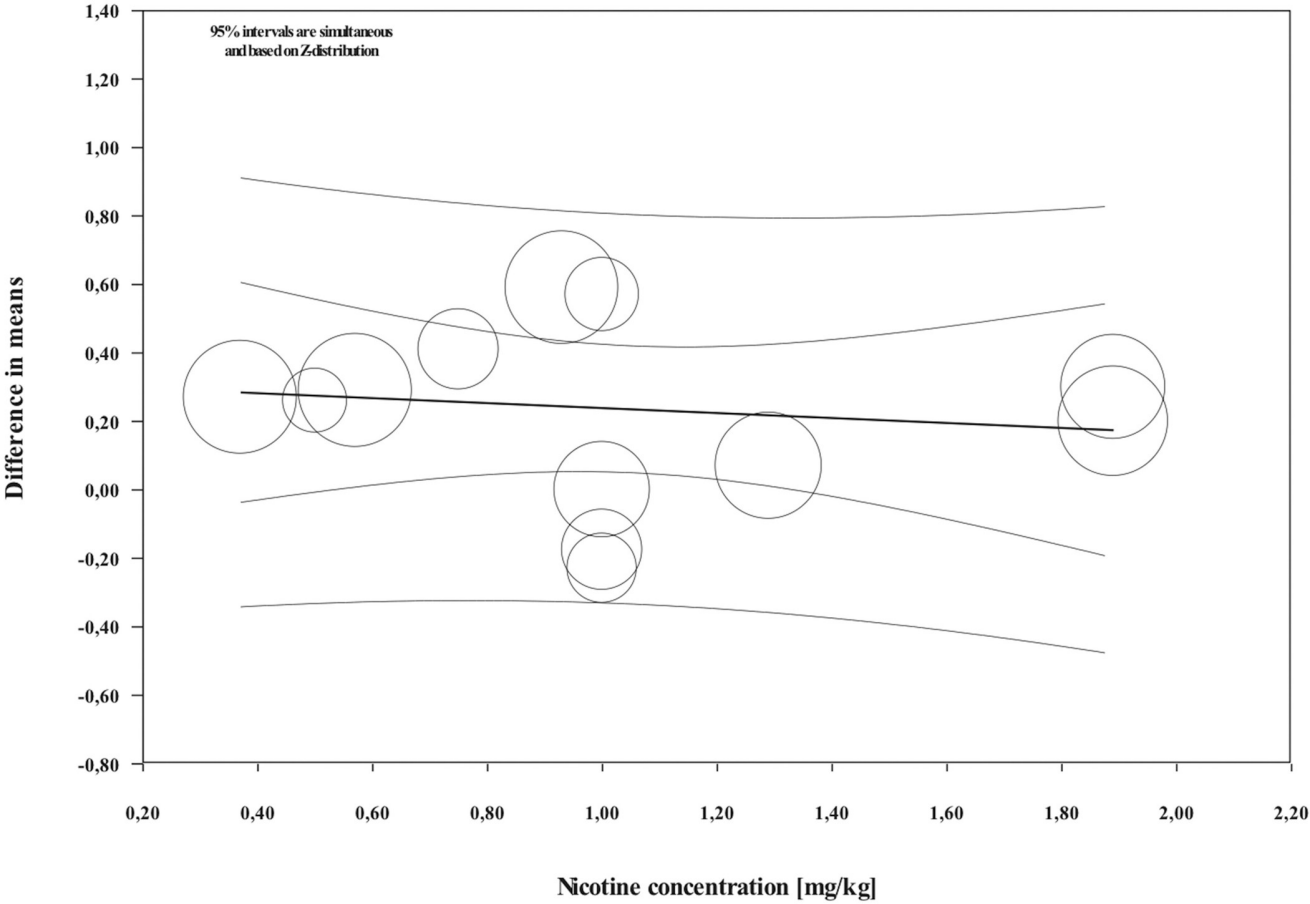
Regression scatterplot of the difference in mesial molar movement between nicotine exposed or not rats, on the predictor of the nicotine concentration (in mg/kg). Confidence intervals and prediction intervals are displayed (inner and outer lines around the regression line).

Overall, during the active period, the amount of mesial molar movement was increased in the nicotine administered rats in comparison with the non-treated animals [WMD: 0.178; 95% CI: 0.083 to 0.274; p = 0.00; I^2^ = 92%] (Fig 2). In the analyses per time point of evaluation, the acceleratory effect after nicotine administration was confirmed only for the observations following 14 days of tooth movement [WMD: 0.317; 95% CI: 0.179 to 0.454; p = 0.00; I^2^ = 94%] (Table 3). In an exploratory meta-regression that included the intercept, the duration of tooth movement (in days) and the concentration of the administered nicotine (in mg/kg), no effect was observed (Table 4 and Figs 3 and 4). Regarding the effect of nicotine administration on tooth movement, the quality of available evidence was considered to be moderate (S3 Table).

**Table 3:** Quantitative synthesis statistics.

		Effect size and 95% confidence interval					Test of null (2-Tail)		Heterogeneity			
Time (d)	N of studies	WMD	SE	Variance	LL	UL	Z-value	P-value	Q-value	df (Q)	P-value	I^2^ [95% UI] (%)
	[datasets]											
2	1	-0.23	0.25	0.06	-0.71	0.25	-0.93	0.35	0.00	0.00	1.00	0.00
7	1	0.07	0.07	0.01	-0.07	0.21	0.95	0.34	0.00	0.00	1.00	0.00
14	4 [8]	0.32	0.07	0.00	0.18	0.45	4.51	0.00	113.98	7.00	0.00	93.86 [90.12–96.18]
28	2	0.09	0.24	0.06	-0.37	0.56	0.40	0.69	5.16	1.00	0.02	80.61 [17.08–95.47]

D: day (s); LL: Lower limit; SE: Standard Error; UL: Upper limit; UI: Uncertainty Interval; WMD: Weighted Mean Difference.

**Table 4:** Main results and statistics for the regression model.

Main results^1^						
Covariate	Coefficient	SE	LL	UL	Z-value	2-sided p-value
**Intercept**	0.2440	0.1929	-0.1341	0.6221	1.26	0.2060
**Duration (in days)**	0.0045	0.0108	-0.0166	0.0256	0.42	0.6742
**Nicotine concentration (in mg/kg)**	-0.0724	0.1379	-0.3427	0.1978	-0.53	0.5933
**Statistics**						
**Test of the model:** Q = 0.35, df = 2, p = 0.8387						
**Goodness of fit:** Tau² = 0.0370, Tau = 0.1924, I² = 93.55%, Q = 139.64, df = 9, p = 0.000						
**Total between-study variance:** Tau² = 0.0295, Tau = 0.1717, I² = 92.18%, Q = 140.73, df = 11, p = 0.000						
**Proportion of total between-study variance explained by the model:** R² analog = 0.00						

^1^Random effects (Method of Moments), Z-Distribution.

LL: Lower limit; SE: Standard Error; UL: Upper limit.

## Discussion

### Summary of evidence

The use of tobacco products is not only relevant to the increased numbers of adult patients seeking orthodontic therapy but also to the older school-aged children that constitute the vast majority of patients under orthodontic treatment [46]. Exposure to nicotine has been reported to affect bone cells and modify the metabolism of the osseous tissue [16–18], with potential effects on the rate of orthodontic tooth movement. Based on the data included in the meta-analysis, nicotine administration increased the rate of movement overall. However, no effect of nicotine concentration or the duration of force application on the rate of movement was observed. Although information from animal studies cannot be fully translated to human clinical scenarios and these analyses are exploratory until more scientific information becomes available, it would be considered safe practice for an orthodontist to be able to identify patients exposed to nicotine and appraise the possible implications.

When the difference in tooth movement between the nicotine administered and the control animals was analysed per time point, an acceleratory effect was observed after 14 days of force application. For this is the time point eight datasets from four studies were analysed and can, thus, be considered the most representative of the analysis. This finding could be explained by the effects of nicotine on bone metabolism described in the literature [47, 48]. Even low concentrations considerably stimulate bone marrow cell proliferation [49]. Nicotine has also been shown to upregulate the formation and differentiation of osteoclasts in general [50, 51] as well as during orthodontic tooth movement [42, 43], even though opposite reports exist as well [40, 44]. *In vitro* compression of human fibroblasts in the presence of nicotine leads to an increase in COX-2, PGE2, IL-6, and Receptor Activator of Nuclear factor-Kappa B Ligand (RANKL) expression, a reduction in OPG expression, and enhanced differentiation of osteoclast-like cells [21]. *In vivo*, Li et al. [39] demonstrated an increase in RANKL levels, which is associated with an accelerated rate of bone turnover [52]. Kirschneck et al. observed that, under the influence of nicotine, osteoclast activity and gene expression of inflammatory and osteoclast markers were significantly increased compared to controls [43].

In addition, high levels of nicotine affect adversely osteoblast proliferation [53] and suppress osteogenesis [54, 55]. Nicotine has also been shown to delay angiogenesis, which in turn delays the formation of the connective tissue and osteogenesis [56]. Although Shintcovsk et al. [38] did not observe an upregulating effect of nicotine administration on osteoclast-like cells and Howship’s lacunae, they found that nicotine affected adversely the bone remodelling mechanism during orthodontic tooth movement by reducing angiogenesis and delaying the collagen maturation process in the developed bone matrix. Furthermore, Araujo et al. [44] demonstrated a reduction in type I collagen after 4 weeks of tooth movement in the nicotine administered group compared to the control animals. The density of the alveolar bone has also been reported to be lower after the administration of nicotine [42]. However, Ferreira and co-workers did not detect a statistically significant difference between animals exposed and non-exposed to cigarette smoke in terms of bone loss and density [45].

In humans, nicotine has been shown to decrease the radiopacity of bone [57] and negatively affect bone mineral density [58]. Such effects have been associated with marked reductions in the volume of trabecular bone, trabecular thickness, mineralizing surface, mineral appositional rate and the rate of formation of bone, coupled with increases in the surfaces covered by the osteoclasts. Overall, nicotine affects adversely the general dynamics of trabecular histomorphometric parameters [17] and the ability of fracture repair [16]. Effects such as those mentioned above could account for the observed acceleratory effect in the rate tooth movement following 14 days of sustained force application.

As far as the other points of observation are concerned, no difference was observed. The meta-regression did not detect an effect of the duration of tooth movement as well. Such findings could be attributed to the scarcity of data pertaining to the other time points and should be considered only as exploratory until further research becomes available. Moreover, tooth movement in rats can be summarized in 3 phases: instantaneous tooth movement, delayed tooth movement and linear phase of tooth movement [59]. After the early response to the orthodontic force that is dependent on the viscoelastic properties of the tissues, a small amount of change occurs in the subsequent delay period due to the hyalinization phenomena. Finally, in the third phase, tooth movement occurs. Based on the above observations, the findings of no difference in the two groups in the early measurements could be anticipated.

In the 28-days measurements, Kirschneck et al. [43] observed an acceleration in the rate of tooth movement whereas Araujo and co-workers [44] a deceleration, leading to a non-significant statistical result after the application of the random effects quantitative synthesis techniques. In general, Araujo et al. [44] was the only study not showing more movement in the nicotine administered group compared to the control after the application of orthodontic force systems. Differences in the experimental setting like animal species, as well as nicotine concentration and duration of administration may have influenced the results [31, 60]. Moreover, differences in measurement methodology could play a role. Kirschneck et al. [43] used CBCT, whereas Araujo et al. [44] used measurements on dental cast to quantify tooth movement.

According to the findings of Sodagar et al. [41] and Bakathir et al. [42], nicotine accelerated the tooth movement in rats in a dose dependent manner. However, in the exploratory meta-regression the concentration of the administered nicotine (in mg/kg) did not show any effect. Once more, differences in the experimental setting like animal species, as well as nicotine route and duration of administration may have influenced the results [60]. The biphasic nature of nicotine effect in bone cells and metabolism may also account for the observed results. Higher nicotine concentrations have predominantly negative effects, whereas low concentrations exert a stimulatory effect in certain cells [16]. Finally, it should not be overlooked that these analyses are exploratory until more scientific information becomes available.

As mentioned previously, it would probably constitute safe practice for orthodontists to detect prospective or existing patients exposed to nicotine. This group of individuals could involve both active and passive smokers, the latter being potentially exposed to significant amounts of nicotine [2]. Prospective orthodontic patients may also use devices and products that contain nicotine [10]. In such individuals, the estimation of the duration of treatment should potentially be modified. In terms of mechanotherapy, it should be taken into consideration that patients may present increased needs for anchorage preparation in cases of space closure. Since it has been reported that orthodontically moved teeth in animals exposed to nicotine exhibit a greater amount of root resorption [37, 39, 43], the magnitude of forces should also be controlled. Furthermore, although no studies evaluating tooth movement after the cessation of the orthodontic forces were located, it could be possible that clinicians might encounter greater difficulty in planning retention in patients that smoke.

It is also important not to overlook the fact that individuals who smoke exhibit increased likelihood to be affected by periodontitis [61]. In such cases, orthodontic mechanical stimuli in conjunction with inflammation of the periodontium might lead to exacerbation of bone resorption and attachment defects [31, 62]. Orthodontic force application in experimental animals has been suggested to lead to a significant increase in nicotine-induced periodontal bone loss [21]; however, opposite reports exist [45]. Other treatment considerations in smokers include the reported lower miniscrew success rates [11–13] and the potential effects on bond strength [14] and biofilm formation [15]. Finally, it should not be neglected that a doctor should always educate the patient in regard to the deleterious effects of smoking and nicotine, as well as encourage smoking cessation [63, 64].

### Strength and limitations

The adherence to established standards regarding methodology can be considered as a strength for the present review. Database search was comprehensive and without restrictions. All steps were performed in duplicate and discussion was used as a means to resolve discrepancies.

Certain limitations in the review are mostly related to the nature or the characteristics of the studies and the information located. The meta-analytical procedures were associated with increased heterogeneity and the meta-regression used to explore whether the variation in molar position was modified by the duration of tooth movement or the concentration of the administered nicotine did not show statistically significant results. Moreover, analyses for “small-study effects”, publication bias and comparisons of the amount of movement between genera/species and studies with different orthodontic mechanics could not be performed due to the lack of adequate information. Thus, the presented statistical elaboration should be considered exploratory and must be viewed with caution until additional research becomes available.

Moreover, currently available data is indirectly related to humans as significant differences exist between them and rats regarding bone physiology [65]. Moreover, the retrieved studies involve the administration of nicotine for different time periods, dosages and routes when compared to the human setting. Such differences may lead to dissimilar effects on nicotine pharmacokinetics and bioavailability. The act of smoking itself, involves a different form of drug administration via the pulmonary rather than the portal or systemic venous circulation [2], resulting in different absorption patterns [66]. Only one study exposed animals to cigarette smoke inhalation, a situation closer to human passive smoking [45]. In such cases, it is possible that some of the chemical compounds included in cigarette smoke other than nicotine, may exert an effect. Additionally, the use of specific modes to induce orthodontic tooth movement further circumscribes the generalizability of the retrieved information to humans [31]. Consequently, it cannot be still clearly determined whether nicotine exposure may affect tooth movement in human clinical scenarios. Nevertheless, we should consider that similar human investigations could exhibit practical obstacles.

### Recommendations for future research

Since nicotine exposure could be relevant to active, as well as passive smokers seeking orthodontic treatment, the need for further research is necessitated. Study designs must be standardized, with low risk of bias and as generalizable as possible, in order to replicate clinical scenarios in daily orthodontic practice.

## Conclusions

Rats administered with nicotine showed overall accelerated rates of orthodontic tooth movement. Although, information from animal studies cannot be fully translated to human clinical scenarios, the orthodontist should be able to identify patients exposed to nicotine and consider the possible implications for everyday clinical practice.

## Supporting information

S1 Checklist PRISMA checklist. (DOC)

S1 Data Data corresponding to Fig 2A from Ferreira et al. (XLSX)

S1 Table Eligibility criteria for the present systematic review. (DOCX)

S2 Table Strategy for database search. (DOCX)

S3 Table Quality of available evidence. (DOCX)
