# Supplementary material for: Effect of nicotine exposure on the rate of orthodontic tooth movement: A meta-analysis based on animal studies
Source: PLoS One. 2021 Feb 17;16(2):e0247011. doi: 10.1371/journal.pone.0247011 (PMC7888643; doi:10.1371/journal.pone.0247011)
Supplement: S1 Table — (DOCX) [file pone.0247011.s003.docx]

**S1 Table.** Eligibility criteria for the present systematic review.

| **Domain** | **Inclusion criteria** | **Exclusion criteria** |
| --- | --- | --- |
| **Participants** | - Healthy, naïve animals (of any age and gender) undergoing any type of orthodontic tooth movement. | - Animals with comorbidities or dietary deficiencies, animals under medication or undergoing any kind of orthodontic tooth movement in conjunction with other interventions such as tooth extraction etc. - Human subjects. |
| **Interventions** | - Nicotine exposure (by any route and dosage). | - Exposure to other substances or nicotine in combination with other substances. |
| **Comparisons** | - Placebo intervention or no intervention. | - Studies without placebo or no intervention groups. |
| **Outcomes** | - Quantitative data, comprehensively reported, regarding the rate of orthodontic tooth movement [i.e. the amount of tooth movement in a specific period of time] measured by various ways [callipers or feeler gauges (directly or on casts), from histological cuts (directly on the optical microscope or from digital photos) or from various kinds of radiographs (lateral cephalometric radiographs, Cone Beam CT, micro-CT, etc.)]. | - Qualitative assessments regarding the rate of orthodontic tooth movement. |
| **Study design** | - Experimental prospective controlled studies (according to the Scottish Intercollegiate Guidelines Network algorithm for classifying study design (available at http://www.sign.ac.uk/ assets/study_design.pdf). | - In vitro, ex-vivo or in silico studies. - Reviews, systematic reviews and meta-analyses. - Less than 5 subjects per group analysed [30]. |
