# Supplementary material for: Effect of nicotine exposure on the rate of orthodontic tooth movement: A meta-analysis based on animal studies
Source: PLoS One. 2021 Feb 17;16(2):e0247011. doi: 10.1371/journal.pone.0247011 (PMC7888643; doi:10.1371/journal.pone.0247011)
Supplement: S2 Table — (DOCX) [file pone.0247011.s004.docx]

**S2 Table.** Strategy for database search.

| **Database [2020 07 31]** | **Search strategy** | **Hits** |
| --- | --- | --- |
| **PubMed** | (nicotine OR smok* OR cigarette* OR tobacco) AND (orthodon* OR “orthodontic force” OR “mechanical force” OR "tooth movement" OR “orthodontic movement” OR “orthodontic anchorage” OR “root resorption”) | **295** |
| **Cochrane Central Register of Controlled Trials** | (nicotine OR smok* OR cigarette* OR tobacco) AND (orthodon* OR “orthodontic force” OR “mechanical force” OR "tooth movement" OR “orthodontic movement” OR “orthodontic anchorage” OR “root resorption”) in Record Title OR (nicotine OR smok* OR cigarette* OR tobacco) AND (orthodon* OR “orthodontic force” OR “mechanical force” OR "tooth movement" OR “orthodontic movement” OR “orthodontic anchorage” OR “root resorption”) in Abstract - (Word variations have been searched) | **117** |
| **Cochrane Database of Systematic Reviews** | (nicotine OR smok* OR cigarette* OR tobacco) AND (orthodon* OR “orthodontic force” OR “mechanical force” OR "tooth movement" OR “orthodontic movement” OR “orthodontic anchorage” OR “root resorption”) in Record Title OR (nicotine OR smok* OR cigarette* OR tobacco) AND (orthodon* OR “orthodontic force” OR “mechanical force” OR "tooth movement" OR “orthodontic movement” OR “orthodontic | **0** |
| **Scopus** | TITLE-ABS((nicotine OR smok* OR cigarette* OR tobacco) AND (orthodon* OR “orthodontic force” OR “mechanical force” OR "tooth movement" OR “orthodontic movement” OR “orthodontic anchorage” OR “root resorption”)) | **159** |
| **Web of Science™** | TOPIC: ((nicotine OR smok* OR cigarette* OR tobacco) AND (orthodon* OR “orthodontic force” OR “mechanical force” OR "tooth movement" OR “orthodontic movement” OR “orthodontic anchorage” OR “root resorption”))  Timespan: All years. Databases: WOS, KJD, RSCI, SCIELO, ZOOREC.  Search language=Auto | **189** |
| **Arab World Research Source** | TI tooth movement AND AB tooth movement | **3** |
| **ProQuest Dissertations and Theses Global** | ti((nicotine OR smok* OR cigarette* OR tobacco) AND (orthodon* OR “orthodontic force” OR “mechanical force” OR "tooth movement" OR “orthodontic movement” OR “orthodontic anchorage” OR “root resorption”)) OR ab((nicotine OR smok* OR cigarette* OR tobacco) AND (orthodon* OR “orthodontic force” OR “mechanical force” OR "tooth movement" OR “orthodontic movement” OR “orthodontic anchorage” OR “root resorption”)) in Full Text | **7** |
