# Supplementary material for: Effect of nicotine exposure on the rate of orthodontic tooth movement: A meta-analysis based on animal studies
Source: PLoS One. 2021 Feb 17;16(2):e0247011. doi: 10.1371/journal.pone.0247011 (PMC7888643; doi:10.1371/journal.pone.0247011)
Supplement: S3 Table — (DOCX) [file pone.0247011.s005.docx]

**S3 Table.** Quality of available evidence.

| - **Quality assessment** | | | | | | - **Effect size** | - **Quality** |
| --- | --- | --- | --- | --- | --- | --- | --- |
| - **Studies** | - **Risk of bias** | - **Inconsistency** | - **Indirectness** | - **Imprecision** | - **Other** | - **WMD and 95% CI** |  |
| **Difference in the amount of tooth movement between nicotine administered and control rats [14 days]** | | | | | | | |
| - 4 studies - [8 datasets] | - Not serious^1^ | - Serious^2^ | - Not serious | - Not serious | - None | - 0.32 mm more in the Nicotine Group - [from 0.18 to 0.45] - p=0.000 | - ⨁⨁⨁◯ - **MODERATE** |

- CI: Confidence Interval; WMD: Weighted Mean Difference
- ^1^No studies with high risk domains were included in the analysis; ^2^Inconsistency was substantial
